# Knowledge and beliefs about dietary inorganic nitrate in a representative sample of adults from the United Kingdom

**DOI:** 10.1017/S1368980024002167

**Published:** 2024-10-28

**Authors:** Alex Griffiths, Evie Grainger, Jamie Matu, Shatha Alhulaefi, Eleanor Whyte, Eleanor Hayes, Kirsten Brandt, John C Mathers, Mario Siervo, Oliver M Shannon

**Affiliations:** 1 School of Health, Leeds Beckett University, Leeds, UK; 2 Human Nutrition & Exercise Research Centre, Centre for Healthier Lives, Population Health Sciences Institute, Newcastle University, Newcastle upon Tyne NE2 4HH, UK; 3 Freeman Hospital, Newcastle upon Tyne, UK; 4 Curtin School of Population Health, Curtin University, Perth, Australia; 5 Curtin Dementia Centre of Excellence, Enable Institute, Curtin University, Perth, Australia

**Keywords:** Dietary nitrate, Nutritional knowledge, Beliefs, Behaviour

## Abstract

**Objective:**

Evaluate knowledge and beliefs about dietary nitrate among United Kingdom (UK)-based adults.

**Design:**

An online questionnaire was administered to evaluate knowledge and beliefs about dietary nitrate. Overall knowledge of dietary nitrate was quantified using a twenty-one-point Nitrate Knowledge Index. Responses were compared between socio-demographic groups.

**Setting:**

UK.

**Participants:**

A nationally representative sample of 300 adults.

**Results:**

Only 19 % of participants had heard of dietary nitrate prior to completing the questionnaire. Most participants (∼70 %) were unsure about the effects of dietary nitrate on health parameters (e.g. blood pressure, cognitive function and cancer risk) or exercise performance. Most participants were unsure of the average population intake (78 %) and acceptable daily intake (83 %) of nitrate. Knowledge of dietary sources of nitrate was generally low, with only ∼30 % of participants correctly identifying foods with higher or lower nitrate contents. Almost none of the participants had deliberately purchased, or avoided purchasing, a food based around its nitrate content. Nitrate Knowledge Index scores were generally low (median (interquartile range (IQR)): 5 (8)), but were significantly higher in individuals who were currently employed *v*. unemployed (median (IQR): 5 (7) *v*. 4 (7); *P* < 0·001), in those with previous nutrition education *v*. no nutrition education (median (IQR): 6 (7) *v*. 4 (8); *P =* 0·012) and in individuals who had heard of nitrate prior to completing the questionnaire *v*. those who had not (median (IQR): 9 (8) *v*. 4 (7); *P* < 0·001).

**Conclusions:**

This study demonstrates low knowledge around dietary nitrate in UK-based adults. Greater education around dietary nitrate may be valuable to help individuals make more informed decisions about their consumption of this compound.

Dietary inorganic nitrate is a water-soluble ion found in both plant- and animal-based foods^(1,2)^. The prime exogenous source of dietary nitrate is vegetables, especially green leafy vegetables and beetroot. However, smaller amounts are also found in processed meat (in which nitrate is used as a preservative), certain fruits, legumes, herbs and water^(1,2)^. Additionally, in recent years, specifically formulated nitrate-containing supplements (e.g. nitrate-rich gels and concentrated beetroot juice ‘shots’) have become commercially available for individuals seeking to increase their intake of dietary nitrate above levels achieved through habitual diet alone^(3,4)^.

Historically, dietary nitrate was considered to be an unwanted food contaminant with potentially deleterious health effects^(5)^. In particular, higher intake of dietary nitrate was viewed as a risk factor for development of certain cancers and infant methemoglobinaemia, which led to recommendations by public health bodies, including the WHO, to limit intake of this compound^(6,7)^. However, with emerging evidence, a new perspective has begun to emerge which suggests that increased intake of nitrate may confer certain health benefits^(4,8,9)^. Indeed, it has now been demonstrated that consumption of dietary nitrate in the form of vegetables or vegetable-derived products can improve markers of cardiovascular (e.g. reduced blood pressure and improved endothelial function)^(10–15)^, brain (e.g. improved cognitive function and modulated cerebral blood flow)^(16–20)^ and oral (e.g. modified oral microbiome and increased resilience against oral acidification) health^(21–24)^. Similarly, nitrate has been shown to improve exercise capacity or performance across a range of population groups^(25–27)^, making it a popular ergogenic aid amongst athletes^(3,28)^.

Research interest in dietary nitrate has increased exponentially in recent years, and in 2019, we demonstrated moderate knowledge of dietary nitrate amongst nutrition professionals, which was greatest in those possessing a PhD (Doctor of Philosophy), indicating some dissemination of nitrate-based knowledge and contemporary research findings amongst nutrition professionals^(29)^. In that study, most nutrition professionals had previously heard of dietary nitrate and believed that it was primarily beneficial, with perceived benefits including improved sports performance and reduced blood pressure. Nutrition professionals also showed good knowledge of dietary sources of nitrate and factors influencing its content in food (e.g. growing conditions and cooking). However, there was limited knowledge in this group about the average population intake and the acceptable daily intake of nitrate.

Presently, there is no information around knowledge or beliefs about dietary nitrate in the general population. It is possible that the general population may have some knowledge about dietary nitrate due to (both positive and negative) media coverage of this compound. Certain population sub-groups, such as recreational or competitive athletes, might also be particularly aware of nitrate given its increasing use in sporting circles as an ergogenic aid^(25)^.

Knowledge and beliefs are important contributors towards behaviour. According to Social Cognitive Theory^(30)^, an individual’s behaviour - in this instance, consumption of dietary nitrate - is influenced by personal factors, an individual’s environment, and their behaviour, with these three factors interacting dynamically and reciprocally^(31)^. Knowledge and beliefs about dietary nitrate could influence an individual’s personal cognitive factors, such as their self-efficacy (i.e. their belief that they can successfully perform a behaviour, such as increasing or decreasing their intake of nitrate-rich foods) and outcome expectations (i.e. the health effects that they expect to occur with consumption of dietary nitrate). A range of environmental factors, including access to information about dietary nitrate, local availability of foods containing nitrate, and community attitudes and behaviours could also influence knowledge and beliefs about nitrate and impact consumption of this compound. A better understanding of knowledge and beliefs about dietary nitrate could therefore be useful in designing interventions which target these factors and consequently influence dietary nitrate intake. For example, identifying gaps in knowledge or misconceptions about dietary nitrate could help to design educational interventions which enhance self-efficacy (e.g. by giving individuals the knowledge and skills needed to adjust their dietary nitrate intake) and influence outcome expectations (e.g. by providing accurate information on the health effects of consuming nitrate-rich foods)^(32)^. Additionally, by exploring what different population sub-groups know and believe about dietary nitrate, it may be possible to personalise these strategies to better meet the needs of different groups. Further benefits of exploring knowledge and beliefs about dietary nitrate include providing information which could be of value to manufacturers and retailers by helping understand whether knowledge and beliefs about dietary nitrate impact purchasing behaviour. In addition, this information could serve as a reference point against which nitrate knowledge and beliefs in the public could be tracked over time or compared against other population groups (e.g. different countries or athletes or clinical populations). Therefore, in this study we aimed to characterise knowledge and beliefs about dietary nitrate in a representative sample of UK adults. We also aimed to identify potential differences in knowledge and beliefs and between different population sub-groups. Our findings are likely to be relevant to researchers, policy makers, public health officials, and food manufacturers or retailers.

## Methods

### Questionnaire development and administration

We used a modified version of the Knowledge of Inorganic Nitrate Dietary Survey (KINDS) questionnaire^(29)^ to characterise knowledge and beliefs about dietary nitrate in the public. This questionnaire was previously developed by our group to evaluate knowledge and beliefs about dietary nitrate amongst nutrition professionals. Modifications were made to ensure appropriateness of language for a non-academic audience, removal of questions regarding biomarkers, metabolic processes and modification of guidelines limiting nitrate intake and the inclusion of questions related to purchasing behaviour. The questionnaire was pilot tested with members of the public to ensure comprehensibility, and modifications were made to the wording and order of questions accordingly. The final questionnaire was sectioned into three parts: (1) demographics (Table 1), (2) knowledge and beliefs (Table 2) and (3) purchasing behaviours (Table 3). Sub-categorising the questionnaire was advocated by participants involved in the pilot testing to improve readability and understanding. A final version of the questionnaire was built using an online survey tool (Online Surveys, Bristol, UK). The questionnaire was administered to a nationally representative sample of 300 participants (matched to the adult (>18 years) UK population regarding age, gender and ethnicity) in December 2022, via Prolific, an online crowd-sourcing platform that provides access to a pool of potential research participants (see^(33)^ for further details). Participants were given modest remuneration (£1·20) for their time, calculated to approximate a living wage (∼£10/h) on a pro-rata basis.

**Table 1.** Participant characteristics

Participant characteristics	*n*	%
Gender		
Male	146	49 %
Female	150	50 %
Other	3	1 %
Do not wish to say	1	0·3 %
Age (years)		
< 20	6	2 %
21–40	123	41 %
41–60	94	31 %
61–80	76	25 %
> 81	1	0·3 %
BMI (kg/m^2^)		
< 18·5	17	6 %
18·5–24·9	142	47 %
25–29·9	102	34 %
> 30	39	13 %
Ethnicity		
White	253	84 %
Asian or Asian British	20	7 %
Black	10	3 %
Mixed	7	2 %
Other	4	1 %
Do not wish to say	6	2 %
Geography		
North East	16	5 %
North West	31	10 %
Yorkshire	26	9 %
East Midlands	24	8 %
West Midlands	27	9 %
South East	50	17 %
South West	39	13 %
London	39	13 %
Scotland	27	9 %
Wales	15	5 %
Northern Ireland	6	2 %
Highest qualification		
O Levels or GCSE	54	18 %
A Level or equivalanet	64	21 %
Vocational qualification	32	11 %
Undergraduate degree	102	34 %
Masters degree	39	13 %
PhD	7	2 %
Other	2	1 %
Nutrition education		
Secondary school	100	33 %
University	17	6 %
Other course	15	5 %
Unsure	13	4 %
No nutrition education	147	49 %
Other	8	3 %
Employment status		
Self-employed	34	11 %
Employed – private sector	83	28 %
Employed – public sector	66	22 %
Student	22	7 %
Unemployed	36	12 %
Retired	59	20 %
Household income		
< £13 000	28	9 %
£13 300–£20 499	23	8 %
£20 500–26 799	45	15 %
£27 000–£35 699	50	17 %
£35 700–£54 000	80	27 %
> £54 000	44	15 %
Do not wish to say	25	8 %
No one in household currently has an income	5	2 %
Exercise		
I do not exercise	89	30 %
1–2 times a week for fitness or recreation	102	34 %
3–5 times a week for fitness or recreation	102	34 %
I take part in competitive sport	7	2 %
Health status		
Heart or circulatory condition	34	11 %
Neurodegenerative disease	5	2 %
Metabolic condition	7	2 %
Kidney disease	2	1 %
Gastrointestinal disorders	17	6 %
Lung disease	16	5 %
Other	38	13 %
No long-term health condition	203	68 %

Percentages for numbers > 1 are rounded to the nearest whole number.

**Table 2.** Knowledge and beliefs in the overall cohort

Question	Overall group response	%
*Have you heard of dietary nitrate?*		
*Yes*	58	19 %
No	198	66 %
Unsure	44	15 %
Do the effects of dietary nitrate on health differ depending upon the type of food it is in?		
Yes	11	4 %
No	6	2 %
Unsure	283	94 %
Does consumption of dietary nitrate from vegetables affect human health?		
Beneficial effect	60	20 %
Harmful effect	7	2 %
Neutral effect	11	4 %
Unsure	222	74 %
Is dietary nitrate from vegetables harmful if consumed in large amounts?		
Yes	41	14 %
No	23	8 %
Unsure	236	79 %
Does consumption of dietary nitrate used as a food additive (e.g. as a preservative in processed meat) affect human health?		
Beneficial effect	11	4 %
Harmful effect	54	18 %
Neutral effect	35	12 %
Unsure	200	67 %
Is dietary nitrate used as a food additive (e.g. as a preservative in processed meat) harmful if consumed in large amounts?		
Yes	93	31 %
No	8	3 %
Unsure	199	66 %
Would consuming too little dietary nitrate have harmful effects? (e.g. such as may occur with a vitamin deficiency)		
Yes	41	14 %
No	32	11 %
Unsure	227	76 %
*For each of the following, please specify if you think it is affected by dietary nitrate from vegetables*		
* Sports performance*		
* Increased*	63	21 %
Decreased	9	3 %
No effect	34	11 %
Unsure	194	65 %
* Blood pressure*		
Increased	38	13 %
* Decreased*	58	19 %
No effect	13	4 %
Unsure	191	64 %
Glucose levels		
Increased	25	8 %
Decreased	25	8 %
No effect	27	9 %
Unsure	223	74 %
Lung function		
Increased	31	10 %
Decreased	7	2 %
No effect	42	14 %
Unsure	220	73 %
Cancer risk		
Increased	43	14 %
Decreased	23	8 %
No effect	27	9 %
Unsure	207	69 %
Cognitive function		
Increased	47	16 %
Decreased	9	3 %
No effect	21	7 %
Unsure	223	74 %
Kidney function		
Increased	41	14 %
Decreased	23	8 %
No effect	12	4 %
Unsure	224	75 %
For each of the following, please specify if you think it is affected by dietary nitrate used as a food additive (e.g. as a preservative in processed meat):		
Sports performance		
Increased	36	12 %
Decreased	28	9 %
No effect	37	12 %
Unsure	199	66 %
Blood pressure		
Increased	65	22 %
Decreased	28	9 %
No effect	18	6 %
Unsure	189	63 %
Glucose levels		
Increased	43	14 %
Decreased	14	5 %
No effect	27	9 %
Unsure	216	72 %
Lung function		
Increased	16	5 %
Decreased	18	6 %
No effect	41	14 %
Unsure	225	75 %
Cancer risk		
Increased	70	23 %
Decreased	9	3 %
No effect	22	7 %
Unsure	199	66 %
Cognitive function		
Increased	27	9 %
Decreased	18	6 %
No effect	31	10 %
Unsure	224	74 %
Kidney function		
Increased	20	7 %
Decreased	31	10 %
No effect	17	6 %
Unsure	232	77 %
*In the general population, how much dietary nitrate does the average person consume each day?*		
< 10 mg/d	22	7 %
11–50 mg/d	28	9 %
*51–200 mg/d*	18	6 %
201–500 mg/d	4	1 %
501–700 mg/d	1	0·3 %
Unsure	227	76 %
*The acceptable daily intake (ADI) of a nutrient or compound is the maximum amount that is safe to consume every day, according to the experts who advise the government. Do you know what the approximate ADI is for dietary nitrate for an average 75 kg adult?*		
Currently no ADI	13	4 %
15 mg/d	16	5 %
*280 mg/d*	21	7 %
1110 mg/d	1	0·3 %
2220 mg/d	0	0 %
Unsure	249	83 %
*For the following foods, do you think they have a low (< 50 mg/100 g food) or high (> 100 mg/100 g food) dietary nitrate content?*		
* Spinach*		
* High*	102	34 %
Low	67	22 %
Unsure	131	44 %
* Sausage*		
High	97	32 %
* Low*	74	25 %
Unsure	129	43 %
* Tomato*		
High	56	19 %
* Low*	92	31 %
Unsure	152	51 %
* Beetroot*		
* High*	95	32 %
Low	68	23 %
Unsure	137	46 %
* Chocolate*		
High	33	11 %
* Low*	97	32 %
Unsure	170	57 %
* Bacon*		
High	109	36 %
* Low*	65	22 %
Unsure	126	42 %
* Lettuce*		
* High*	55	18 %
Low	105	35 %
Unsure	140	47 %
* Radish*		
* High*	67	22 %
Low	85	28 %
Unsure	148	49 %
*Which of the following factors do you think may affect the dietary nitrate content of food?*		
* If it has been cooked*		
* Yes*	137	46 %
No	27	9 %
Unsure	136	45 %
* Season it was produced in*		
* Yes*	66	22 %
No	67	22 %
Unsure	167	56 %
* Soil conditions*		
* Yes*	146	49 %
No	20	7 %
Unsure	134	45 %
* Fertiliser*		
* Yes*	161	54 %
No	14	5 %
Unsure	125	42 %
* How the food is stored*		
* Yes*	88	29 %
No	56	19 %
Unsure	156	52 %
* If the food is pickled*		
* Yes*	84	28 %
No	43	14 %
Unsure	173	58 %
* Does drinking water contain dietary nitrate?*		
No	57	19 %
* Yes, <50 mg/l*	34	11 %
Yes, between 51 and 100 mg/l	1	0·3 %
Yes, between 101 and 200 mg/l	0	0 %
Yes, between 201 and 300 mg/l	0	0 %
Yes, but don’t know how much	35	12 %
Unsure	173	58 %
Do you use an antibacterial mouthwash?		
Yes	126	42 %
No	152	51 %
Unsure	22	7 %
*Do you think using mouthwash would influence the effects of dietary nitrate?*		
*Yes*	6	2 %
No	75	25 %
Unsure	219	73 %

Questions contributing towards the nitrate knowledge index, alongside the answer deemed to be the correct response, are identified in italics.

**Table 3.** Purchasing behaviour in the overall cohort

Question	Overall group response	%
Have you ever specifically chosen a food because it contains dietary nitrate?		
Yes	2	1 %
No	285	95 %
Unsure	13	4 %
Have you ever avoided choosing a food because it contains dietary nitrate?		
Yes	14	5 %
No	269	90 %
Unsure	17	6 %
How frequently do you take nutritional supplements?		
I do not take nutritional supplements	137	46 %
Every day	83	28 %
On most days	45	15 %
At least once in most weeks	20	7 %
Approximately once every month	8	3 %
Approximately once every year	7	2 %
Have you ever taken a supplement to increase your intake of dietary nitrate?		
No	282	94 %
Yes, I have done this but only once	0	0 %
Yes, on most days	1	0·3 %
Yes, at least once in most weeks	0	0 %
Yes, approximately once every month	0	0 %
Yes, approximately once every year	0	0 %
Unsure	17	6 %
How willing would you be to purchase a supplement containing dietary nitrate if scientific evidence that it could improve cardiovascular health (e.g. lower blood pressure or reduce risk of a heart attack) was approved for marketing?		
Very unlikely	22	7 %
Unlikely	36	12 %
Neutral	57	19 %
Likely	111	37 %
Very likely	39	13 %
Unsure	35	12 %
How willing would you be to purchase a supplement containing dietary nitrate if scientific evidence that it could improve cognition or brain (e.g. improve brain function or reduce risk of dementia) was approved for marketing?		
Very unlikely	19	6 %
Unlikely	16	5 %
Neutral	65	22 %
Likely	118	39 %
Very likely	60	20 %
Unsure	22	7 %
How willing would you be to purchase a supplement containing dietary nitrate if scientific evidence that it could improve metabolic health (e.g. help control blood glucose or reduce risk of diabetes) was approved for marketing?		
Very unlikely	25	8 %
Unlikely	28	9 %
Neutral	74	25 %
Likely	104	35 %
Very likely	49	16 %
Unsure	20	7 %
How willing would you be to purchase a supplement containing dietary nitrate if scientific evidence that it could improve exercise performance (e.g. allow you to exercise for longer or perform better in a competition) was approved for marketing?		
Very unlikely	48	16 %
Unlikely	65	22 %
Neutral	77	26 %
Likely	61	20 %
Very likely	28	9 %
Unsure	21	7 %
Would you be more or less likely to purchase a supplement containing dietary nitrate if the nitrate came from vegetables rather than other sources?		
Much less likely to purchase	4	1 %
Less likely to purchase	10	3 %
Neutral	100	33 %
More likely to purchase	107	36 %
Much more likely to purchase	39	13 %
Unsure	40	13 %
Would you be more or less likely to purchase a vegetable which was deliberately produced to have a high nitrate content than one which wasn’t?		
Much less likely to purchase	14	5 %
Less likely to purchase	31	10 %
Neutral	133	44 %
More likely to purchase	33	11 %
Much more likely to purchase	9	3 %
Unsure	80	27 %
Would you be more or less likely to purchase a processed meat product which had been deliberately produced without nitrate, compared with one which included nitrate?		
Much less likely to purchase	32	11 %
Less likely to purchase	27	9 %
Neutral	102	34 %
More likely to purchase	39	13 %
Much more likely to purchase	6	2 %
Unsure	94	31 %
If you wanted to increase your daily nitrate intake, would you rather do this via consumption of nitrate supplement or by increasing your intake of nitrate-rich foods?		
Supplement	49	16 %
Food	186	62 %
Neutral	19	6 %
Unsure	46	15 %
If you wanted to increase your intake of nitrate specifically before an exercise session or competition, would you rather do this via consumption of a specific nitrate supplement or by increasing your intake of nitrate-rich foods?		
Supplement	92	31 %
Food	110	37 %
Neutral	33	11 %
Unsure	65	22 %
Would you be interested or willing to learn about the impacts of dietary nitrate on your health?		
Yes, I want to know	146	49 %
It would be useful, but I am not interested in this	78	26 %
I don’t mind	52	17 %
No, I don’t want to know	24	8 %

### Calculation of nitrate knowledge index

Similar to our previous research within nutrition professionals^(29)^, a twenty-one-point index was derived to provide a quantitative measure of overall knowledge about dietary nitrate. We identified questions where there was unambiguous evidence for a correct answer. In such cases, participants were awarded one point for correct responses and zero points for incorrect responses (italicised in Table 2). Questions where current evidence is inconclusive or for which no correct response is available were excluded from the Index. Data from recent reviews and an expert consensus statement on nitrate informed decision making on correct or incorrect responses^(4,10,25,34,35)^.

### Statistical analysis

Data analyses were conducted using the Statistical Package for the Social Sciences (SPSS) version 28, whilst figures were created using GraphPad Prism. Differences in knowledge and beliefs about dietary nitrate between different population sub-groups were compared using the *χ* ^2^ test, whilst the Mann–Whitney *U* test was used to compare scores on the Nitrate Knowledge Index between population sub-groups. These population sub-groups were defined by age (younger (< 40 years) *v*. older (≥ 40 years)), gender (male *v*. female), ethnicity (white *v*. other), education level (lower (GCSE, A Level, vocational, other) *v*. higher (undergraduate degree, Master’s degree or PhD)), employment status (employed and self-employed *v*. other), household income (lower (< £35 700) *v*. higher (≥ £35 700)), BMI (BMI; < 25 kg/m^2^ *v*. ≥ 25 kg/m^2^), exercise level (lower (do not exercise) *v*. higher (other)), level of nutrition education (lower (no nutrition education, unsure and other) *v*. higher (secondary school level of nutrition education and above)), and whether participants had previously heard of nitrate (heard of nitrate *v*. have not heard of nitrate). *P* < 0·05 was accepted for statistical significance. Raw data are available in an online repository (https://data.ncl.ac.uk/) and can be accessed by contacting the authors.

## Results

### Participant characteristics

A total of 300 participants completed the questionnaire (Table 1). There was a similar percentage of male (49 %) and female (50 %) participants within the study, distributed across all geographical areas of the UK. Participants were mostly white (84 %), the most common age group was 21–40 years (41 %) and the most common BMI range was18·5–24·9 kg/m^2^ (47 %). Most commonly, participants reported exercising 1–2 (34 %) or 3–5 (34 %) times per week. An undergraduate degree was the most common qualification held (34 %). The participants reported mixed levels of nutrition education, with around half reporting no nutrition education (49 %) and a third (33 %) reporting only basic nutrition education at secondary school.

### Overall knowledge and beliefs about dietary nitrate

An overview of participant responses is provided in Tables 2 and 3. Overall, only 19 % of participants had heard of dietary nitrate prior to completing the questionnaire, compared with 66 % who had not heard of nitrate and 15 % who were unsure if they had heard of nitrate before.

#### Health effects of nitrate

Participants were generally unsure as to whether dietary nitrate from vegetables (74 %) or as a food additive (67 %) would affect human health. Similarly, most of the participants were unsure as to whether dietary nitrate from vegetables or used as a food additive would affect sports performance (vegetables: 65 % unsure, food additive: 66 % unsure), blood pressure (vegetables: 64 % unsure, food additive: 63 % unsure), glucose levels (vegetables: 74 % unsure, food additive: 72 % unsure), lung function (vegetables: 73 % unsure, food additive: 75 % unsure), cancer risk (vegetables: 69 % unsure, food additive: 66 % unsure), cognitive function (vegetables: 74 % unsure, food additive: 74 % unsure) and kidney function (vegetables: 75 % unsure, food additive: 77 % unsure).

#### Nitrate sources and acceptable daily intake

Knowledge around dietary nitrate intake was typically poor. Most participants (76 %) were unsure of the average population intake of dietary nitrate and the nitrate acceptable daily intake (83 %). Knowledge of dietary sources of nitrate was generally poor, with ∼20–30 % of participants correctly identifying spinach, beetroot, lettuce and radish as high in nitrate and ∼20–30 % of participants correctly identifying sausage, tomato, chocolate and bacon as low in nitrate. Around half of the participants were aware that the dietary nitrate content of food is influenced by cooking (46 %), soil conditions (49 %) and fertiliser (54 %), but a smaller percentage were aware of the influence of season it was produced in (22 %), how it was stored (29 %) and if the food was pickled (28 %). Participants were mostly unsure as to whether drinking water contains dietary nitrate (58 %) and whether the use of mouthwash would influence the effects of dietary nitrate (73 %).

#### Purchasing behaviour

Most participants had not chosen (95 %) to purchase foods because they contained dietary nitrate. Similarly, most participants had not avoided (90 %) purchasing foods because they contained nitrate and had not consumed supplements to increase their intake of dietary nitrate (94 %). Around half of all participants reported that they would be likely or very likely to purchase a supplement containing dietary nitrate if scientific evidence demonstrated it could improve cardiovascular health (50 %), cognition (59 %) and metabolic health (51 %), but a smaller percentage were likely or very likely to purchase a nitrate-containing supplement to improve exercise performance (30 %). Participants reported that they were more likely or much more likely to purchase a supplement containing dietary nitrate if it came from a vegetable rather than other sources (49 %), whereas they were generally neutral with regard to whether they would purchase a vegetable (44 %) or processed meat products (34 %) which were deliberately produced to have a high dietary nitrate content. Interestingly, most participants reported that they would rather increase their intake of dietary nitrate via consumption of nitrate-rich foods (62 %) rather than supplements (16 %). Responses were more varied in relation to consuming nitrate prior to exercise, with 37 % and 31 % of participants preferring to increase dietary nitrate intake via food and supplements, respectively. There was a willingness to learn about the impact of dietary nitrate on health, with 49 % of participants stating they would like to know more.

### Differences in knowledge and beliefs in different population groups

There were some significant differences in knowledge and beliefs about dietary nitrate between different participant sub-groups, which are highlighted below and in online Supplementary Table 1. However, most participants (regardless of population sub-group) responded ‘unsure’ to the majority of questions.

#### Age

Knowledge and beliefs around the health effects of dietary nitrate consumption as a food additive differed according to age. Specifically, participants aged ≥ 40 years were more likely to believe that nitrate provided as a food additive is harmful compared with those < 40 years (25 % *v*. 9 %; *P =* 0·002). Those aged ≥ 40 years were also more likely to perceive *excess* consumption of dietary nitrate as a food additive as harmful, compared with those < 40 years (39 % *v*. 21 %; *P =* 0·004). Beliefs around the physiological effects of dietary nitrate consumption were generally consistent between older and younger adults, although participants aged ≥ 40 years were more likely to believe that nitrate intake from vegetables increased cancer risk (≥ 40 years: 17 %, < 40 years: 11 %, *P* = 0·006). A larger proportion of those aged ≥ 40 years believed that the way in which food was stored could influence its nitrate content compared with those aged < 40 years (33 % *v*. 25 %; *P =* 0·02).

With regard to purchasing behaviour, a greater percentage of participants aged ≥ 40 years compared with < 40 years had not specifically chosen a food to increase dietary nitrate intake (98 % *v*. 91 %; *P =* 0·008). Similarly, those aged ≥ 40 years were marginally more likely to have avoided a specific food because it contains dietary nitrate than those aged < 40 years (7 % *v*. 2 %; *P =* 0·019). A greater percentage of participants aged ≥ 40 years had not taken a supplement to increase dietary nitrate compared with those aged < 40 years (97 % *v*. 90 %; *P =* 0·03). Interestingly, participants aged < 40 years were more likely to state that they would rather increase their dietary nitrate intake prior to exercise or competition via a supplement (43 % *v*. 22 %; *P* < 0·001), whereas participants aged ≥ 40 years had a greater preference to consume whole foods to increase their nitrate intake prior to exercise compared with those < 40 years (44 % *v*. 26 %).

#### Gender

A greater proportion of male compared with female participants believed that the effects of dietary nitrate on health differ depending on the type of food it is in (7 % *v*. 1 %; *P =* 0·012). Beliefs around the physiological effects of dietary nitrate consumption were generally consistent between those of different genders, although males were more likely than females to believe that dietary nitrate consumption as a food additive increased exercise performance (17 % *v*. 7 %; *P =* 0·038). A greater proportion of male participants believed that the use of antibacterial mouthwash would influence the effects of dietary nitrate compared with female participants (4 % *v*. 0 %; *P =* 0·041). Male participants were more likely or much more likely than female participants to purchase a nutritional supplement containing dietary nitrate to improve exercise performance (36 % *v*. 24 %; *P* < 0·001) and were also more likely or much more likely to purchase a vegetable which was deliberately produced to have a high nitrate content (19 % *v*. 9 %; *P =* 0·024).

#### Ethnicity

Beliefs around the physiological effects of dietary nitrate from vegetables were generally consistent between those of different ethnicities. However, a greater percentage of participants from ethnic minority groups believed that dietary nitrate from vegetables either had either no effect on cancer risk (19 % *v*. 7 %) or decreased risk (13 % *v*. 7 %; *P =* 0·016) compared with those of white ethnicity. A larger percentage of participants from ethnic minority groups compared with white ethnicity perceived dietary nitrate as a food additive to decrease sports performance (21 % *v*. 7 %; *P =* 0·016), increase blood pressure (36 % *v*. 19 %; *P =* 0·051) and increase glucose levels (28 % *v*. 12 %; *P =* 0·043). Participants from ethnic minority groups also showed some indications of better knowledge about nitrate on specific questions, compared with white participants. For example, a greater percentage of participants from ethnic minority groups minority compared with white participants were aware that lettuce is high in nitrate (32 % *v*. 16 %; *P =* 0·008) and correctly identified the nitrate content of drinking water (26 % *v*. 9 %; *P =* 0·009). Participants from ethnic minority groups were also more likely than those of white ethnicity to want to learn more about the impact of dietary nitrate on health (68 % *v*. 45 %; *P =* 0·037).

#### Education

A greater proportion of participants with higher compared with lower-level educational qualifications believed that the effects of dietary nitrate on health did not differ depending on the type of food it is in (4 % *v*. 0 %; *P =* 0·039). Beliefs around the physiological effects of dietary nitrate consumption were generally consistent between those with different qualifications, although participants with a higher-level qualification were more likely than those with a lower-level qualification to believe that dietary nitrate consumption via a food additive increased cognitive function (12 % *v*. 6 %; *P =* 0·036).

#### Employment status

There was a greater percentage of employed compared with non-employed participants who believed that spinach (41 % *v*. 23 %; *P =* 0·006), tomato (24 % *v*. 10 %; *P =* 0·01), beetroot (38 % *v*. 21 %; *P =* 0·008) and radish (28 % *v*. 14 %; *P =* 0·015) are high in nitrate, and that bacon is low in nitrate (27 % *v*. 13 %; *P =* 0·01). A greater percentage of employed compared with non-employed participants believed that antibacterial mouthwash does not impact the effects of dietary nitrate in the body (31 % *v*. 15 %; *P =* 0·008). A greater percentage of employed compared with non-employed participants stated that they would rather use a dietary supplement to increase their nitrate intake prior to exercise (34 % *v*. 25 %; *P =* 0·034).

#### Income

Participants with higher compared with lower income were more likely to want to use a dietary supplement to increase their nitrate intake prior to exercise (36 % *v*. 25 %), whereas those with a lower income were more likely to want to consume food to increase dietary nitrate prior to exercise or competition (46 % *v*. 28 %; *P =* 0·002).

#### BMI

Beliefs around the physiological effects of dietary nitrate consumption were generally consistent between those with different BMI, although those with a BMI < 25 kg/m^2^ compared with a BMI ≥ 25 kg/m^2^ were more likely to believe that dietary nitrate intake from vegetables (18 % *v*. 6 %; *P =* 0·008) and as a food additive (28 % *v*. 14 %; *P =* 0·031) increases blood pressure.

#### Exercise level

A greater percentage of those engaging in exercise compared with no exercise perceived spinach to be high in nitrate (38 % *v*. 25 %; *P =* 0·01). A greater percentage of participants not engaging compared with engaging in exercise perceived no effect of cooking (17 % *v*. 6 %; *P =* 0·005) or season (29 % *v*. 19 %; *P =* 0·046) on the nitrate content of food. Those who engage in exercise were more likely to want to learn more about dietary nitrate than those who do not (54 % *v*. 36 %; *P =* 0·035).

#### Nutrition education

Those with a higher compared with lower level of nutrition education were more likely to have heard of dietary nitrate (27 % *v*. 13 %; *P =* 0·002). A greater percentage of participants with a higher compared with lower level of nutrition education believed that dietary nitrate from vegetables increases sports performance (28 % *v*. 16 %; *P =* 0·047) and cognitive function (23 % *v*. 10 %; *P =* 0·02), and that dietary nitrate as a food additive increases cognitive function (14 % *v*. 5 %; *P =* 0·018). A greater percentage of participants with a higher compared with lower level of nutrition education perceive soil content to influence the nitrate content of foods (53 % *v*. 45 %; *P =* 0·007).

#### Prior knowledge of nitrate

Knowledge or awareness of nitrate prior to undertaking this survey had the biggest impact on responses to the questionnaire. Participants who had heard of nitrate prior to undertaking this survey were more likely (than those who had not heard of nitrate) to believe that the health effects of nitrate differ depending upon the type of food it is in (14 % *v*. 2 %; *P* < 0·001), believing that nitrate from vegetables is beneficial for health (45 % *v*. 14 %; *P* < 0·001), but that consumption of nitrate as a food additive is harmful for health (40 % *v*. 10 %; *P* < 0·001) (for beliefs about on individual health outcomes, see online Supplementary Table 1). Participants who had heard of nitrate were generally (although not always) better at identifying foods with higher and lower nitrate content. These individuals were also more likely to believe that the nitrate content of food could be influenced by whether it had been cooked (55 % *v*. 45 %; *P* = 0·031), the soil conditions (71 % *v*. 42 %; *P* < 0·001), use of fertiliser (74 % *v*. 51 %; *P* = 0·001), how it is stored (41 % *v*. 26 %; *P* = 0·008) and if the food is pickled (40 % *v*. 24 %; *P* = 0·019).

Participants who had heard of nitrate were more likely to correctly identify that drinking water typically contains < 50 mg/l nitrate (26 % *v*. 8 %; *P* < 0·001). Participants who had heard of nitrate more commonly stated that they were ‘very likely’ to purchase a supplement containing nitrate if scientific evidence that it could improve cardiovascular health was approved for marketing (21 % *v*. 11 %; *P* = 0·042) and that they were ‘much more likely’ to purchase a supplement where the nitrate came from vegetable sources (26 % *v*. 9 %; *P* = 0·007). Similarly, participants who had heard of nitrate more commonly stated that they were ‘less likely’ to purchase a vegetable deliberately produced to have a high nitrate content (17 % *v*. 8 %; *P* = 0·024) and ‘more likely’ to purchase a meat product produced without nitrate (29 % *v*. 9 %; *P* < 0·001) than those who had not heard of nitrate. Sub-group analyses (analyses by participant characteristics) were run separately in participants who had or had not heard of nitrate as exploratory analyses and are presented in online Supplementary Table 1.

### Nitrate knowledge index

**Fig. 1:**
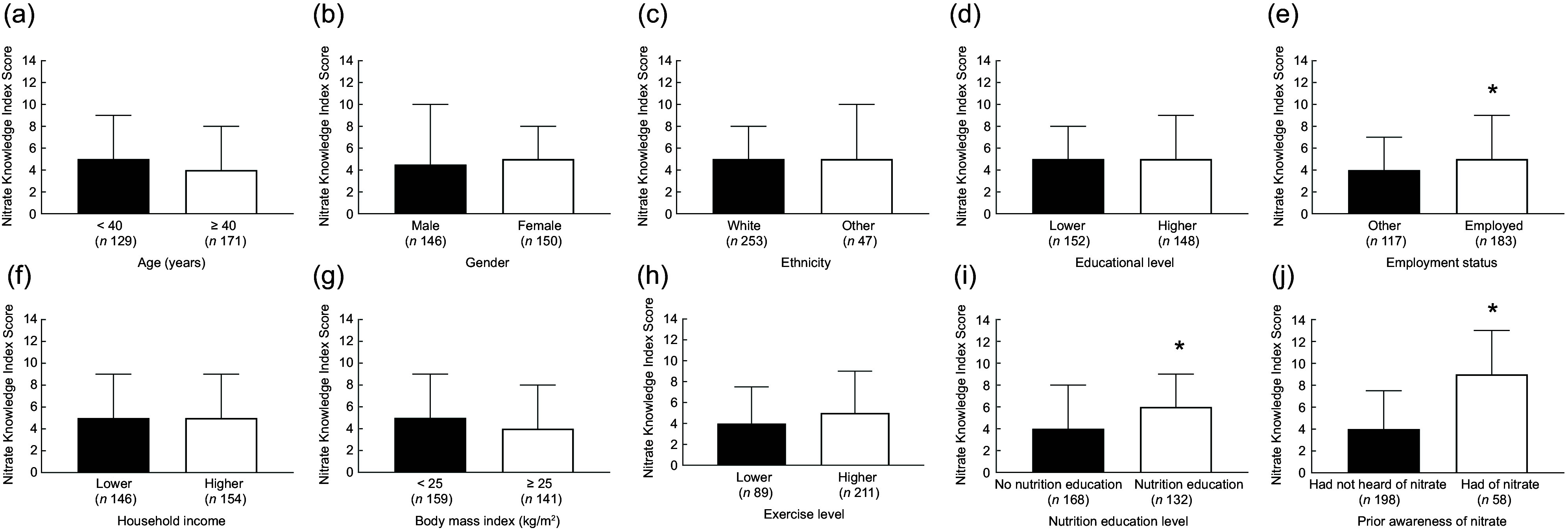
Nitrate Knowledge Index scores in different sociodemographic groups. Analyses were stratified by age (panel A; younger (< 40 years) *v*. older (≥ 40 years)), gender (panel B; male *v*. female), ethnicity (panel C; white *v*. other), education level (panel D; lower (GCSE, A Level, vocational, other) *v*. higher (undergraduate degree, Master’s degree or PhD)), employment status (panel E; employed and self-employed *v*. other), household income (panel F; lower (<£35 700) *v*. higher (≥£35 700)), BMI (panel G; <25 kg/m^2^ *v*. ≥25 kg/m^2^), exercise level (panel H; lower (do not exercise) *v*. higher (other)), level of nutrition education (panel I; lower (no nutrition education, unsure and other) *v*. higher (secondary school level of nutrition education and above)), and prior knowledge of nitrate (panel J; had not heard of nitrate *v*. heard of nitrate). Data presented are median (IQR). * = significant difference (*P* < 0·05) between groups. Individuals who were employed, with higher nutrition education, and who had heard of nitrate prior to completing the questionnaire showed significantly greater knowledge of dietary nitrate IQR, interquartile range.

Overall knowledge about dietary nitrate was quantified using a Nitrate Knowledge Index. In the entire cohort, the median (interquartile range; IQR) score for the Nitrate Knowledge Index was 5 (8) out of a possible twenty-one points. Knowledge Index stratified by participants demographics is presented in Fig. 1. There were no significant associations between age (*P =* 0·558), gender (*P =* 0·558), ethnicity (*P =* 0·52), level of qualification (*P =* 0·978), income (*P =* 0·535), BMI (*P =* 0·246) or exercise (*P =* 0·377) and the Nitrate Knowledge Index. However, there was a significant association between employment status and the Nitrate Knowledge Index, with those in employment achieving a higher score compared with those not in employment (Nitrate Knowledge Index (median (IQR)): 5 (7) *v*. 4 (7); *P =* 0·0007). Nutrition education was also significantly associated with the Nitrate Knowledge Index score, with those with a higher nutrition education achieving a higher score than those with a lower nutrition education (Nitrate Knowledge Index (median (IQR)): 6 (7) *v*. 4 (8); *P =* 0·012). In addition, participants who had previously heard of nitrate before completing the questionnaire had significantly higher Nitrate Knowledge Index scores than those who had not previously heard of nitrate (median (IQR): 9 (8) *v*. 4 (7); *P* < 0·001).

## Discussion

In this study, we set out to evaluate knowledge and beliefs about dietary inorganic nitrate in a representative sample of adults from the UK. We found that knowledge of dietary nitrate was generally poor, with only one fifth of the participants having heard of this compound prior to completing the questionnaire. In comparison, in a previous study from our group evaluating knowledge and beliefs about dietary nitrate amongst 125 UK-based nutrition professionals, > 70 % of participants had previously heard of dietary nitrate^(29)^. We evaluated overall knowledge about dietary nitrate via a twenty-one-point Nitrate Knowledge Index. Median score for the Knowledge Index was five out of twenty-one in the entire cohort, reflecting typically poor knowledge about this compound. In comparison, in our previous study in nutrition professionals in which nitrate knowledge was evaluated via a twenty-three-point Index (the higher total score available was due to the addition of two questions related to metabolism of nitrate, which was not considered to be relevant in this investigation), the group median score was 12.

In the current study, a minority of participants were able to correctly identify health effects associated with nitrate. Indeed, there appeared to be a general inability to distinguish between scientifically proven effects of nitrate (e.g. blood pressure reduction^(10,14,36)^ or improved exercise performance^(25–27)^) and other physiological effects with limited or no supporting evidence (e.g. lung or kidney function). This suggests poor dissemination of current nitrate-related knowledge outside of scientific communities. Only around one-third of participants were able to correctly identify foods with a higher or lower dietary nitrate content and therefore would struggle to make informed decisions about the purchase of foods containing this compound. A larger percentage of participants (∼50 %) were able to correctly identify some factors impacting the nitrate content of food (e.g. cooking, soil conditions and and use of fertiliser). However, this could reflect general knowledge of factors the impact the nutritional properties of food, rather than knowledge specific to nitrate. In contrast, in our previous study in nutrition professionals, knowledge about the physiological effects of nitrate, food sources of this compound and factors affecting its content was much greater (typically ∼50 % of nutrition professionals gave correct responses for questions on these factors).

Previous research suggests that overall nutritional knowledge in both students^(37)^ and adults^(38,39)^ in the UK population is typically low, which mirrors the findings seen here for a specific nutritional compound dietary nitrate. There is some previous evidence to suggest variations in overall nutritional knowledge between different socio-demographic groups, with men typically having lower nutritional knowledge than women and individuals with lower education levels or a lower socio-economic status also possessing lower levels of nutritional knowledge^(38)^. Although we found some variation in response to individual questions within these groups, we did not find any overall difference in Nitrate Knowledge Index scores by gender, overall education levels or household income (our closest proxy for socioeconomic status). However, we found that individuals who reported some previous nutrition education scored on average two points higher on the twenty-one-point Nitrate Knowledge Index than those without any previous nutrition education. This is broadly consistent with the findings from our previous investigation in which we found that individuals with higher level nutritional qualifications (possession of a Master’s or PhD) typically had greater knowledge of nitrate than individuals with lower level qualifications (undergraduate degree)^(29)^. Interestingly, in the current study, individuals who were employed or self-employed also had greater (median 1 point higher) scores on the Nitrate Knowledge Index than individuals who were retired, unemployed or students. This agrees with some previous research in Australian adults, which showed greater overall nutritional knowledge in employed *v*. unemployed adults^(40)^. It is possible that employed individuals may have had greater nutrition education or exposure to relevant information as part of their employment. The greatest differences in Nitrate Knowledge Index scores were observed between those had *v*. had not heard of dietary nitrate. Specifically, those who had heard of nitrate scored on average five points higher on the Nitrate Knowledge Index score than those who had not heard of dietary nitrate. Differences in knowledge were particularly apparent when identifying the health effects of dietary nitrate, the dietary nitrate content of different foods and factors that influence the dietary nitrate content of foods.

Our study provides new information on whether an individual’s knowledge or beliefs about dietary nitrate impacts, or could impact, their behaviour when purchasing foods. Almost all participants reported that they had not deliberately purchased, or avoided purchasing, a food because of its nitrate content. This suggests that simply marketing a food as higher or lower nitrate (which is sometimes the case for processed meat products which have been deliberately prepared without nitrate due to perceived health risks of this compound) may not impact consumer behaviour. Similarly, most participants had not previously purchased a nitrate-rich supplement to increase their intake of this compound, although around half of the participants suggested they would be likely or very likely to do this if there was evidence to suggest this could have cardiovascular, metabolic or cognitive benefits. Therefore, if such claims were approved for use in marketing products, they could have important implications for the sale of such supplements. It is interesting to note that most participants suggested that they would rather increase their intake of nitrate via consumption of nitrate-rich foods rather than supplements. This information could help with the design of interventions and public health campaigns to augment nitrate intake by the public if a sufficient evidence-base was to emerge to support the health benefits of such an approach. Potential advantages of increasing nitrate intake via food *v*. supplements, previous highlighted by our group^(3)^, include lower cost, greater variety and provision of fibre which is often lacking in nitrate-based supplements but can have health benefits^(41,42)^. Nevertheless, whilst foods are often preferred to supplement (by both individuals and nutritionists or dietitians), there may still be a potential role for supplements under certain circumstances (e.g. athletes looking to consume a high nitrate bolus pre-competition)^(43)^. It is also relevant to note that we did not directly enquire about participants views on concentrated beetroot juice, which is a commonly used nitrate supplement, but could potentially be perceived more favourably than nitrate supplements in tablet or pill form.

Given the typically poor knowledge of dietary nitrate and associated health benefits, it may be pertinent to develop and/or optimise public health education strategies. This is particularly relevant given the high rates of ^(44)^ and dementia^(45)^ in the UK, and the evidence that dietary nitrate may improve markers of cardiovascular^(10–14)^ and brain^(16–19)^ health (contrasting the historical view that nitrate is a potentially harmful compound to be eradicated from the diet). Although improved nutritional knowledge does not guarantee behaviour change, it can contribute towards and facilitate such changes^(46,47)^. Given widespread use of social media websites or applications, such channels may represent an easy-to-use, low-cost, direct way for nutritional educators to reach a relevant audience^(48)^. Such strategies could be tailored for different socio-demographic groups to maximise impact and knowledge dissemination.

From an international perspective, the findings of limited knowledge about dietary nitrate in the public may only be relevant to those countries who practice similar legislation to the UK, such as countries within the European Union. Here, mention of any food-related health benefit in a marketing context is banned, unless the corresponding health claim has received official approval^(49)^. Neither the UK nor the European Union has approved any nitrate-related health claims, and, as such, there is no legal marketing of this nature. In other regions of the world, such as the USA, where the legislation is less restrictive^(50)^, companies are allowed to advertise the health effects of dietary supplements such as nitrate (e.g. via television and social media). It is possible that knowledge about dietary nitrate could be greater in those regions, and future research is required to measure this.

### Strengths and limitations

This study includes a relatively large sample, similar to or greater than the number of participants in previous investigations into nutritional knowledge^(29,40,51,52)^. The dataset produced could serve as a reference point for future investigations into nitrate knowledge in different populations or to evaluate change in knowledge in the public over time. We matched our participant characteristics to the wider UK population by age, gender and ethnicity. However, we were unable to ensure all characteristics of our participants were representative of the UK population, including education and employment status, both of which can influence nutritional knowledge and habits^(40)^. Considering this was a nutrition-based questionnaire, it is possible that respondents may have had a greater interest in nutrition compared with non-respondents. However, as the key finding ultimately reflects a limited knowledge of nitrate, the prior interests of participants are unlikely to have significantly affected the general ‘take home’ message from this study. Another limitation of the study is that few participants (*n* 58) had heard of dietary nitrate prior to completing the questionnaire. It is, therefore, possible that some responses from individuals who had not heard of nitrate were educated guesses based on general nutritional knowledge and beliefs. Additional analysis demonstrated that those that had heard of nitrate performed significantly better than those that had not heard of nitrate. A final strength of this questionnaire is that it underwent considerable pilot testing with members of the public, which maximised comprehensibility^(53)^.

### Conclusion

This study provides new insight into knowledge and beliefs about dietary nitrate in the general population of the UK. Overall, results show that knowledge about dietary nitrate is poor, and notably lower than previously observed for nutrition professionals. Greater education around this compound may be valuable to help individuals make more informed decisions about their consumption of nitrate-containing foods. This could occur as part of broader efforts to increase nutritional knowledge in the population, which could be an important strategy to mitigate risk of diet-associated diseases including, diabetes, cancer and dementia^(54–56)^.

## Supporting information

Griffiths et al. supplementary material Griffiths et al. supplementary material

## Supplementary material

For supplementary material accompanying this paper visit https://doi.org/10.1017/S1368980024002167
